# Requirement of RIZ1 for Cancer Prevention by Methyl-Balanced Diet

**DOI:** 10.1371/journal.pone.0003390

**Published:** 2008-10-13

**Authors:** Wenyun Zhou, Sergio Alonso, Daisaku Takai, Shelly C. Lu, Fumiichiro Yamamoto, Manuel Perucho, Shi Huang

**Affiliations:** 1 Cancer Research Center, The Burnham Institute for Medical Research, La Jolla, California, United States of America; 2 Gastrointestinal and Liver Diseases, USC Research Center for Liver Diseases, USC-UCLA Research Center for Alcoholic Liver and Pancreatic Diseases, Keck School of Medicine, University of Southern California, Los Angeles, California, United States of America; 3 Institute of Biomedical Sciences, Center for Evolutionary Biology, Fudan University, Shanghai, China; Karolinska Institutet, Sweden

## Abstract

**Background:**

The typical Western diet is not balanced in methyl nutrients that regulate the level of the methyl donor S-adenosylmethionine (SAM) and its derivative metabolite S-adenosylhomocysteine (SAH), which in turn may control the activity of certain methyltransferases. Feeding rodents with amino acid defined and methyl-imbalanced diet decreases hepatic SAM and causes liver cancers. RIZ1 (PRDM2 or KMT8) is a tumor suppressor and functions in transcriptional repression by methylating histone H3 lysine 9.

**Methodology/Principal Findings:**

Here we show that a methyl-balanced diet conferred additional survival benefits compared to a tumor-inducing methyl-imbalanced diet only in mice with wild type RIZ1 but not in mice deficient in RIZ1. While absence of RIZ1 was tumorigenic in mice fed the balanced diet, its presence did not prevent tumor formation in mice fed the imbalanced diet. Microarray and gene expression analysis showed that, unlike most of its related enzymes, RIZ1 was upregulated by methyl-balanced diet. Methyl-balanced diet did not fully repress oncogenes such as c-Jun in the absence of RIZ1. Higher RIZ1 activity was associated with greater H3 lysine 9 methylation in RIZ1 target genes as shown by chromatin immunoprecipiation analysis.

**Conclusions/Significance:**

The data identify RIZ1 as a critical target of methyl-balanced diet in cancer prevention. The molecular understanding of dietary carcinogenesis may help people make informed choices on diet, which may greatly reduce the incidence of cancer.

## Introduction

The typical Western diet is linked to a third of all cancer deaths in the United States [1]. The diet is rich in meat and low in vegetables and fruits. It is not balanced in methyl nutrients or low in folic acid. Dietary nutrients, and their metabolic intermediates and products, directly influence the activity of many cellular enzymes. One class of such enzymes is SAM-dependent methyltransferases, a broad group of enzymes that have one property in common, the use of S-adenosylmethionine (SAM) as methyl group donor. The cellular level of SAM depends on dietary intake of methyl group donors, such as methionine, folic acid, vitamin B6, B12, and choline. Some methylation reactions are inhibited by low level SAM or high level of the product inhibitor S-adenosylhomocysteine (SAH). Methyl imbalanced diet that is low in folic acid, methionine, or choline is known to lower SAM level and SAM/SAH ratio.

Feeding rodents with amino acid defined and methyl-imbalanced diet decreases hepatic SAM and causes liver cancers [2], [3], [4]. The molecular mechanisms underlying the relationship between diet and cancer remain poorly understood. We have previously proposed that methyl-balanced diet prevents cancer by activating the histone lysine methyltransferase (KMT) class tumor suppressors such as RIZ1 (PRDM2 or KMT8) [5], [6]. The RIZ1 tumor suppressor functions in transcriptional repression by methylating histone H3 lysine 9 [7], [8], [9]. Here, we determined whether RIZ1 may be a critical target of methyl balanced diet in cancer prevention. We also performed microarray and gene expression analysis to study the effect of diet on RIZ1 and other genes. The effect of diet on RIZ1 methylation enzyme activity was analyzed by chromatin immunoprecipiation assay. The results suggest that RIZ1 is regulated by diet and may be a critical target of methyl-balanced diet in cancer prevention.

## Results

**Figure 1:**
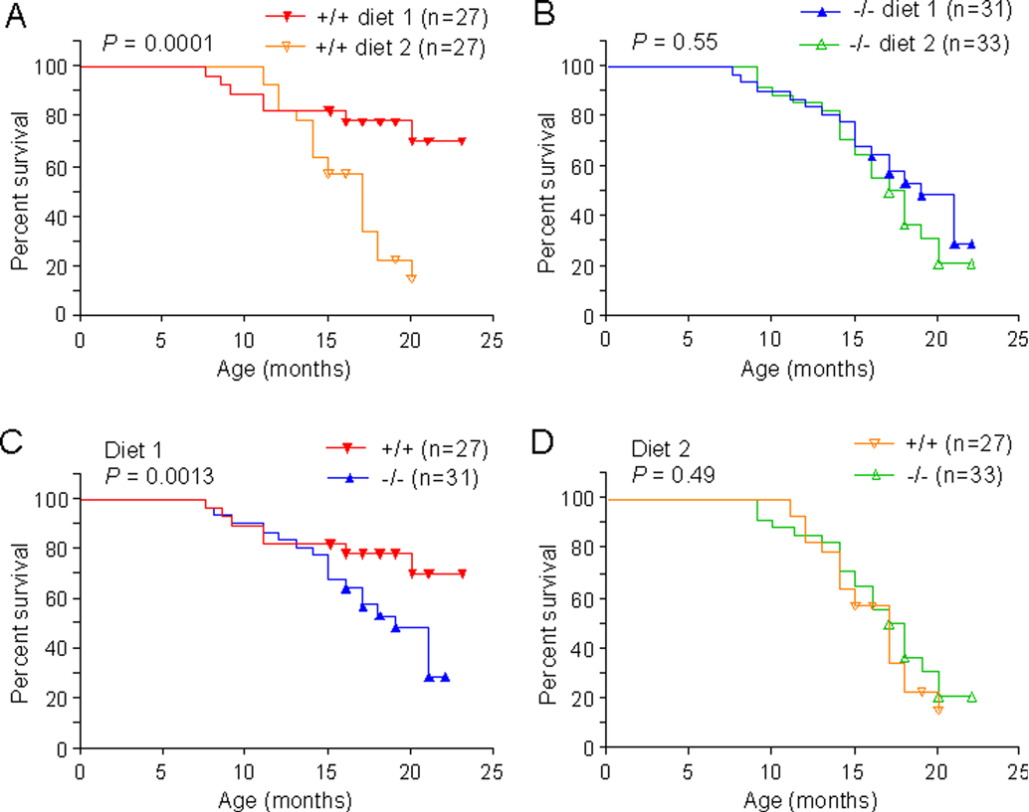
Survival of RIZ1 wild type and mutant animals on diet 1 versus diet 2. A. The viability of RIZ1 wild type animals on diet 1 versus diet 2. B. The viability of RIZ1 mutant animals on diet 1 versus diet 2. C. The viability of RIZ1 wild type and mutant animals on diet 1. D. The viability of RIZ1 wild type and mutant animals on diet 2. Most of the dead or moribund animals that were suitable for autopsy analysis were found to have tumors. The graph was drawn with the Prism statistics software program (GraphPad Software) based on the Kaplan-Meyer theory. *P* values were calculated by Fisher's exact test (2 tailed) using the survival rate at ages 20 to 22 months.

We compared RIZ1 mutant and wild type mice on a methyl-balanced diet (diet 1), versus an imbalanced diet lacking methionine and choline (diet 2). The methyl-imbalanced diet 2 (see Supplementary Table S1) is well known to lower hepatic SAM and cause liver cancers in rodents [2], [3], [4]. Thus, this methyl-imbalanced diet caused liver tumors and decreased survival compared with the methyl-balanced diet (Figure 1A). Most of the dead or moribund animals that were suitable for autopsy analysis were found to have hepatocarcinomas. In contrast, in the absence of wild type RIZ1, there was no difference in survival regardless of diet (Figure 1B). These RIZ1 knockout animals developed mostly hepatocarcinomas regardless of diet. Therefore, while the balanced diet 1 conferred additional survival benefits compared to the imbalanced diet 2 in mice with wild type RIZ1, it failed to do so in mice deficient in RIZ1.

The data also shows that, consistent with previous work [7], RIZ1+/+ mice had lower mortality and tumor incidence than RIZ1−/− mice when fed methyl-balanced diet 1 (Figure 1C). However, when fed imbalanced diet 2, RIZ1+/+ mice showed similar mortality as RIZ1−/− mice (Figure 1D). Thus the tumor suppressor function of RIZ1 is dependent on a methyl-balanced diet. The similar survival rates of RIZ1-deficient and wild type animals on diet 2 (Figure 1D) also suggests that the capacity of diet 1 to confer additional survival benefits compared to diet 2 in RIZ1 wild type (Figure 1A) but not in RIZ1-deficient animals (Figure 1B) is not because of the trivial reason that the RIZ1-deficient animals may be too sick in general to respond to diet 1.

**Figure 2:**
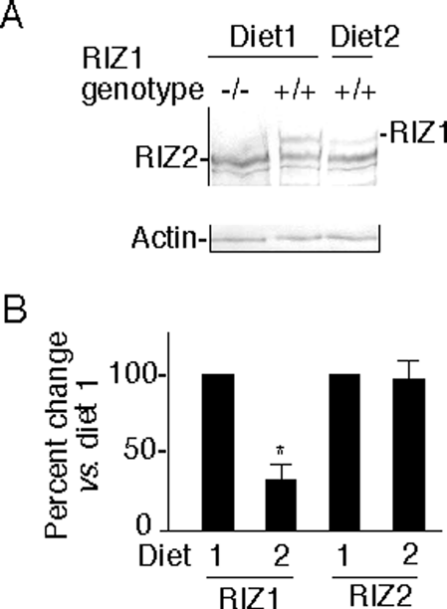
Regulation of RIZ1 protein expression by diet. A. Whole cell extracts of liver from animals on diet 1 and diet 2 for two months were analyzed by Western blot analysis using an antibody that reacts with both RIZ1 and RIZ2 proteins. Western blot using beta-actin antibody served as loading control. B. Quantification of protein levels by densitometry analysis. Data are the means±SD of 4 animals per subgroup. *P = 0.012 (Student's t-test, 2 tailed).

To determine the effects of diet on RIZ1, we examined RIZ1 gene expression in the liver target tissue using quantitative RT-PCR and Western blot analysis. RIZ1 mRNA level was downregulated 4.2 fold after treatment with diet 2 for 2 months (Supplementary Table S2). The downregulation was not evident at 1 month on diet 2 but became obvious at 2, 4, and 6 months (Supplementary Table S2). The downregulation of RIZ1 by the methyl-imbalanced diet was confirmed by western blot analysis (Figure 2). In contrast to RIZ1, the shorter RIZ2 protein that lacks the PR/SET domain [10] was not significantly affected by diet.

We also examined other methyltransferases and related enzymes by quantitative RT-PCR and DNA microarray analysis. A total of 25 histone methyltransferases were examined, including at least one enzyme specific for each of the amino acid residues that are known to be methylated. None of these enzymes, except RIZ1, was significantly downregulated (>2 fold) at 2 months of diet 2 treatment (Supplementary Table S2).

To further determine whether RIZ1 expression is sensitive to SAM levels, we used the MATA1 knockout mice model [11]. These animals have lower (3 to 4 fold) hepatic SAM and also develop liver cancers. Quantitative RT-PCR analysis of animals at 4.5–5.5 months of age showed that RIZ1 expression in wild type livers (n = 6) was 2.0 fold higher than in the MATA1 knockouts (n = 5, P = 0.03, Student's t test, 2 tailed), comparable to the fold reduction for the same aged wild type mice on diet 2 (Supplementary Table S2).

We next examined whether RIZ1 target genes were regulated by diet. DNA microarray analysis of livers of RIZ1 wild type animals at 2 months of diet treatment revealed a list of 1636 genes that were affected by diet by more than 2 fold (Supplementary Table S3). DNA microarray analysis of livers of RIZ1 knockout animals identified 97 putative RIZ1 target genes showing more than 2 fold difference between wild type and knockout (Supplementary Table S4). Of these, 29 were also present in the list of genes regulated by diet, indicating a significant enrichment of RIZ1 target genes in the list of diet-regulated genes (29/97 versus 1636/48000, *P*<0.0001, Chi squared test, 2 tailed). The genes of interest that were upregulated by both RIZ1 knockout and methyl-imbalanced diet include c-Jun, c-Fos, and Ctgf. Some of these genes might play a direct role in liver cancers (i.e., c-Jun) [12]. The effect of RIZ1 knockout or diet on the expression of these genes was confirmed by quantitative RT-PCR analysis (Table 1). The results suggest that downregulation of RIZ1 by diet 2 was associated with deregulation of RIZ1 target genes.

**Table 1:** Quantitative RT-PCR analysis of genes that were upregulated by RIZ1 deficiency or by diet 2 treatment.

Genes	KO vs. WT	Diet 2 vs. Diet 1	
		2 months	1 month
Abcc4	2.1	13.1	10.2
Alas1	4.0	2.3	1.3
Ctgf	2.2	2.4	2.3
Fos	2.4	5.0	4.7
Gdf15	2.3	2.7	3.7
Jun	2.0	8.0	2.6
Mthfd1l	1.6	5.1	3.0

Fold changes in liver RNA expression of 7 putative RIZ1 target genes in RIZ1 knockout versus wild type animals were shown. Also shown are fold changes in liver RNA expression of 7 putative RIZ1-target genes in RIZ1 wild type animals on diet 2 versus diet 1 after either 1 month or 2 months of diet treatment. Data represent means of at least 3 animals per subgroup. *P*<0.05 (Student's t-test, 2 tailed) for all data set except Alas1 at 1 month diet treatment.

Since RIZ1 expression was not significantly altered by diet 2 at 1 month treatment (Supplementary Table S2), any expression changes of the target genes of RIZ1 at 1 month diet treatment may reflect changes in RIZ1 activity rather than in expression level. We selected seven RIZ1 target genes and determined their expression levels at either 1 month or 2 months diet treatment. As shown in Table 1, six of the seven genes were found upregulated by diet 2 at 1 month diet treatment. The data suggest that diet 2 caused deregulation of RIZ1 target genes before significantly decreasing RIZ1 expression levels.

**Figure 3:**
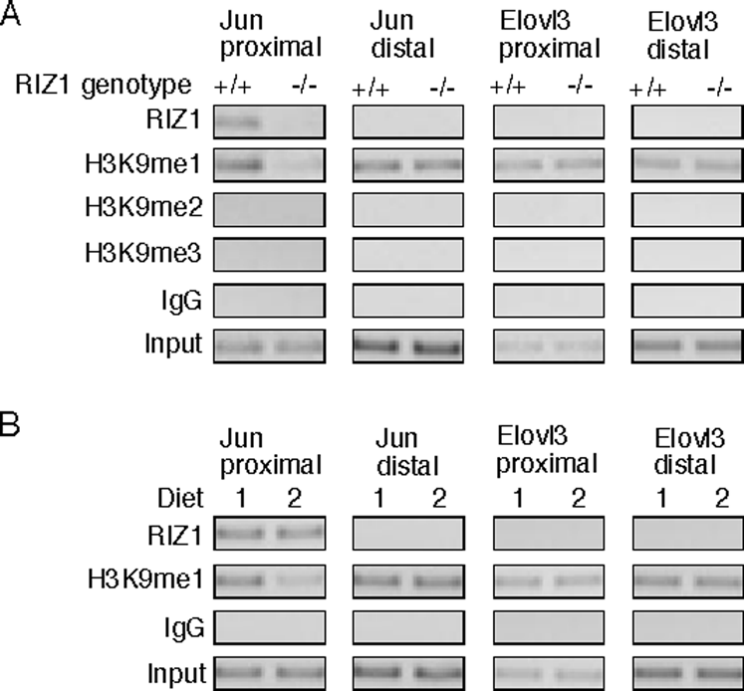
Regulation of histone methylation on RIZ1 target genes. A. Soluble chromatin was prepared from livers of RIZ1 wild type and knockout animals. Immunoprecipitation was performed using the indicated antibodies. DNA was amplified using primer sets that cover the proximal or the distal promoter regions of c-Jun and Elovl3 gene. B. Soluble chromatin was prepared from livers of wild type animals on either diet 1 or diet 2 for 1 month. Immunoprecipitation was performed using the indicated antibodies. DNA was amplified using primer sets that cover the proximal or the distal promoter regions of c-Jun and Elovl3 gene. One representative data set is shown out of four experiments performed.

We next used chromatin immunoprecipitation (ChIP) assay to examine changes in histone methylation on RIZ1 target genes as a result of decreased RIZ1 activity. We used a RIZ1-specific antibody ab9710 (Abcam) that does not react with RIZ2 (see Supplementary Figure S1 for a Western blot of RIZ1 by this antibody). As shown in Figure 3A, the c-Jun proximal promoter was bound by RIZ1 and showed higher levels of H3 lysine 9 monomethylation in RIZ1 wild type versus knockout livers. RIZ1 did not bind to the distal region of the c-Jun promoter or the promoter of Elovl3 gene that was not repressed by RIZ1. At 1 month diet 2 treatment, H3K9me1 of c-Jun promoter was decreased even though RIZ1 binding was not changed, suggesting again that RIZ1 activity was decreased even when RIZ1 expression was at normal levels (Figure 3).

We also examined overall DNA methylation changes in response to methyl-imbalanced diet in DNA from livers of animals at 15 months of treatment. While we found that diet 2 decreased overall DNA methylation (Supplementary Figure S2), we did not find changes in DNA methylation (either hyper or hypo methylation) in individual random CpG rich regions (Supplementary Figure S3) using the technique of *Not1-Mse1* MS-AFLP (methylation sensitive amplified fragment length polymorphism) [13]. By Western blot analysis, we did not find significant changes (>2 fold) in methylation of H3K9me1, H3K9me2, H3K9me3, H4K20me1, and H4K20me2 in the liver of animals that were on diet 2 for six months (data not shown).

## Discussion

Since only RIZ1 and 4 small molecule methyltransferases (GNMT, GAMT, NNMT, and TEMT) were found downregulated by diet 2 in a non-biased screening using both DNA microarray and RT-PCR methods (see Supplementary Table S2), we examined whether these 5 enzymes shared some common properties that could explain their sensitivity to diet. We found from the literature Km values for SAM for 3 of the 4 small molecule methyltransferases and all three turned out to have high Km value (>49 µM) (see Supplementary Table S5). For the large number of methyltransferases that were not regulated by diet, we identified Km values for 7 of them and all 7 have Km values lower than 10 µM (see Supplementary Table S5). Thus, there is a significant association between high Km for SAM and downregulation by diet 2 (*P* = 0.0083, Fisher's exact test, 2 tailed).

Hepatic SAM concentration is ∼60 µM and methyl-imbalanced diet typically causes a 1.5 to 3 fold drop in SAM levels [3], [14], which would be still high enough to allow near maximum activity for most methyltransferases with low Km for SAM but would significantly inhibit those enzymes with high Km values. The preferential downregulation by diet 2 of the methyltransferases with high Km for SAM may reflect a feedback inhibition on their own gene expression by unliganded or unused enzymes. The canonical SET domain shared by most KMTs has low Km for SAM [15]. But RIZ1 may be an exception since its SET domain is different from others [16], [17].

Numerous effects of methyl-imbalanced diet, such as fat accumulation, protein kinase C activation, abnormal cell turnover, oxidative stress, low SAM/SAH ratio, DNA hypomethylation, and uracil incorporation into DNA or mutation, are prevented by a methyl-balanced diet. In addition, a methyl-imbalanced diet affects the expression of numerous genes as shown here. Our observation that deficiency of a single gene may neutralize the cancer preventive benefit of a methyl-balanced diet shows that at least one of the numerous effects of such diet may play a rate-limiting role in the cancer prevention process.

From all these observations it can be postulated that the molecular pathway of carcinogenesis by methyl-imbalanced diet may be the following: a methyl-imbalanced diet — chronic low SAM/SAH ratio in most cells of a diet-responsive target tissue —inhibition and downregulation of RIZ1 in most cells of the target tissue — upregulation of prosurvival and progrowth oncogenes such as c-Jun in most cells of the target tissue — stochastic accumulative genetic and epigenetic changes in a single cell — clonal proliferation of the single cell — cancer. In this multi-cell origin model of cancer initiation [6], inactivation of RIZ1 and upregulation of some oncogenes such as c-Jun in most cells of a diet-responsive tissue may not be sufficient to cause clonal proliferation but may allow a large pool of less than fully normal cells to be more prone to clonal proliferation in response to stochastic strong oncogenic mutations in a single cell. In contrast, fully normal cells may undergo senescence or cell death in response to oncogenic mutations [18]. Thus, a rate-limiting step in cancer prevention may be the maintenance of RIZ1 activity in most cells of a target tissue [6].

## Materials and Methods

### Animals, diets, and tissues

RIZ1 knockout mice in 129Sv/C57Bl6 background were crossed to 129Sv mice for 8 generations to produce RIZ1 knockout mice in 99.625% 129Sv background. These RIZ1−/− mice were then crossed with p53+/− 129Sv mice (from Jackson Laboratory) to generate RIZ1+/−p53+/− mouse in 129Sv background. Animals of the following genotypes were maintained for experimental use: RIZ1−/−p53+/− and RIZ1+/+p53+/−. The introduction of p53 heterozygous mutation was intended to shorten the latency of tumor development in the RIZ1 knockout mice [7]. Male animals of each genotype were randomized into two groups of ∼30 each. Starting at about 4 weeks of age, all animals were fed with a methyl-balanced and amino acid-defined diet or diet 1. After on this diet for one week, one group of animals continued to stay on diet 1 for the remaining period of the experiment. The other group were fed with methionine and choline deficient diet or diet 2. The compositions of the diets (see Supplementary Table S1) were essentially the same as those commonly used in the field [2], [3], [4]. Diets were stored at 4°C and given ad libitum with biweekly replacement. Animal growth was followed by monthly body weight measurement for upto 15 months. No significant difference in body weight was noted for different genotypes. Animals on diet 2 showed reduced (15% less at 15 months of age) body weight compared to animals on diet 1. This slight effect of diet 2 on body weight was similar to what others have found [2]. Kaplan-Meyer survival curves were plotted using the Prism statistics program (GraphPad software). The liver tissues were fixed for histological analyses or snap frozen in liquid nitrogen and stored at −80°C until used for biochemical analyses. Tissues fixed in 10% formalin were routinely processed for paraffin embedding, sectioned, and stained with hematoxylin and eosin. For measurements of dietary effects on gene expression and histone methylation, additional animals were fed diet 1 or 2 for 1 to 6 months before being sacrificed for tissue collection.

Ethical approval for all works on animals was obtained from the Animal Research Committee of The Burnham Institute for Medical Research.

### Western blotting

Tissues were homoginized in RIPA buffer (150 mM NaCl, 1% Nonidet P-40, 0.25% Na-deoxycholate, 1 mM EDTA, 50 mM Tris-HCl, pH 7.4, plus proteinase inhibitors). The whole lysates were then mixed with SDS gel loading buffer followed by SDS gel fractionation and western blotting. RIZ antibody was rabbit serum KG7.1S against RIZ1 aa 245–573 that reacts with both RIZ1 and RIZ2, as described previously (available from Abcam ab3790) [19]. For western blot against RIZ1 but not RIZ2, we used the abcam RIZ1 antibody ab9710. Antibodies for methylated histones were from Abcam and Upstate.

### Quantitative RT-PCR

Total RNAs were extracted from tissues using the MagNa Lyser Green Beads (Roche) and the RNAmini kit (Qiagen). Quantitative RT-PCR analyses (SYBR green) were performed using the Mx3000P QPCR system of Stratagene. All gene specific primers were confirmed to give a single band of expected size. Cyclophilin A (PPIA) gene served as a control for RNA amount. This gene was not regulated by diet or RIZ1 knockout as indicated by microarray analysis.

### DNA microarray analysis

cDNAs synthesized from total RNAs were hybridized with Sentrix Mouse-6 Expression BeadChip from Illumina containing 48,000 gene arrays. Data were obtained from replicate biological samples. Data normalization was performed using cubic spline normalization.

### ChIP analysis

Rabbit antibody specific for RIZ1 but not RIZ2 from Abcam (ab9710) was used for ChIP analysis of RIZ1 binding to target genes. Antibodies for methylationed histones were from Abcam (ab9045 for H3K9me1, ab8898 for H3K9me3, ab9051 for H4K20me1) and Upstate (07-212 for H3K9me2). Liver tissues (∼30 mg per antibody immunoprecipitation) were chopped to pieces using razor blades and cross linked in 1% formaldehyde at room temperature for 15 min. Chromatin was fragmented to an average size of 700 bp using a Misonix XL2020 sonicator. The ChIP assay kit from Upstate was used. For PCR, we used the following primer pairs: 5′-AAATCTCTGGTTTCCAGGTACAGC-3′ (−801 to −777) and 5′- GAGAAAGGGCTGAATGATCTGAGT-3′ (−595 to −571) for c-Jun proximal promoter region, and 5′-GTGTGGGGGTAGAGGAGTGA-3′ (−4337 to −4317) and 5′- AGTGTCACTGGACCCTCACC -3′ (−4128 to −4108) for c-Jun distal region. For PCR of Elovl3 gene promoter, we used the primer pairs 5′-CCCCATTTTTCTCTCCAACA-3′ (−768 to −748) and 5′-CCAAGCTGGACCATAAGGAA-3′ (−578 to −558) for the Elovl3 promoter region, and 5′-CCAGGCTGTCCTGAAACTAATTCT-3′ (−8325 to −8301) and 5′-CTGTAGACCAGGTGACTGCAAACT-3′ (−8132 to −8108) for the Elovl3 distal region.

### DNA methylation analysis

Genomic DNAs were extracted from tissues using standard procedures. Analysis of methylation in CpG rich regions by *Not1-Mse1* MS-AFLP method was as described previously [13]. Genomic methyl-cytosine content was assayed by the methylation enzyme Sss1 method [20].

## Supporting Information

Table S1 Composition of the amino acid-defined and methyl-balanced basal diet (Teklad Product No. TD 99366). This is referred as Diet 1. The methyl-imbalanced diet or diet 2 formulation ((Teklad Product No. TD 01513) is the same as diet 1 except that it contains 9.0 g/Kg DL-Homocystine and lacks methionine and choline bitartrate.
(0.05 MB DOC)

Table S2 Quantitative RT-PCR and microarray analysis of some histone methyltransferases and related enzymes. Wild type male animals were treated with diet 1 or diet 2 (starting from 3–4 weeks of age) for 1, 2, 4, and 6 months before RNA extraction from livers. Levels of 25 histone methyltransferases and 8 other related enzymes were determined by either quantitative RT-PCR or microarray or both, and the ratio (fold change) Diet 1 versus Diet 2 calculated. Data from quantitative RT-PCR represent means of at least 3 animals per subgroup. *P<0.05 Diet 1 vs. Diet 2 (Student's t-test, 2 tailed). Histone methyltransferases that are not listed here did not show significant expression in the liver as revealed by DNA microarray analysis. Data from microarray analysis represent means of 2 animals per subgroup. DNA microarray analysis revealed downregulation by diet 2 of four small molecule methyltransferases, GNMT, GAMT, NNMT, and TEMT, which were subsequently confirmed by quantitative RT-PCR.
(0.08 MB DOC)

Table S3 Genes regulated by diet as revealed by DNA microarray analysis. Wild type mice were fed with either diet 1 or diet 2 for two months before their livers were collected for RNA extraction. Data represent means of 2 animals per subgroup. Only genes showing more than 2 fold changes are listed.
(0.85 MB XLS)

Table S4 Genes regulated by RIZ1 knockout as revealed by DNA microarray analysis. The RNAs used for analysis were from wild type and knockout mice of four months of age on balanced diet. Data represent means of 2 animals per subgroup. Genes in bold were also regulated by diet. Only genes showing more than 2 fold changes are listed.
(0.06 MB XLS)

Table S5 Methyltransferases with high Km value for SAM were downregulated by methyl-imbalanced diet. The Km value for SAM and Ki value for SAH are shown for various methyltransferases. The ratio (fold changes) Diet 1 vs. Diet 2 in expression levels of the methyltransferases was calculated from quantitative RT-PCR. Data represent means of at least 3 animals per subgroup at 2 months of diet treatment. *P<0.05 Diet 1 vs. Diet 2 (Student's t-test, 2 tailed).
(0.03 MB DOC)

Figure S1 Western blot analysis confirming recognition of RIZ1 but not RIZ2 by the Abcam RIZ1-specific antibody ab9710. Total protein extracts of livers from either RIZ1 wild type or knockout animals were resolved by SDS gel followed by western blot using RIZ1 antibody ab9710. Equal loading was confirmed by western blot using beta-actin antibody.
(0.07 MB PDF)

Figure S2 DNA hypomethylation in mice fed with diet 2. Total genomic DNAs were isolated from livers of mice on either diet 1 or diet 2 for 15 months. The methylation levels of these DNAs were determined by the Sss1 enzyme assay. Data are the means+SD of 3 animals per subgroup. *P = 0.03 (Student's t-test, 2 tailed).
(0.02 MB PDF)

Figure S3 Analysis of methylation changes in CpG rich regions of the genome by MS-AFLP. A. Genomic DNA isolated from MCF7 cells that were either treated or not treated with the demethylating agent Aza-C were used as a positive control for the MS-AFLP method. B. The genomic DNAs used for analysis were from brain (B) and liver (L) tissues of mice on either diet 1 or diet 2 for 15 months.
(1.49 MB PDF)
