# Supplementary material for: Requirement of RIZ1 for Cancer Prevention by Methyl-Balanced Diet
Source: PLoS One. 2008 Oct 13;3(10):e3390. doi: 10.1371/journal.pone.0003390 (PMC2559864; doi:10.1371/journal.pone.0003390)
Supplement: Table S1 — Composition of the amino acid-defined and methyl-balanced basal diet (Teklad Product No. TD 99366). This is referred as Diet 1. The methyl-imbalanced diet or diet 2 formulation ((Teklad Product No. TD 01513) is the same as diet 1 except that it contains 9.0 g/Kg DL-Homocystine and lacks methionine and choline bitartrate. (0.05 MB DOC) [file pone.0003390.s001.doc]

|  | g/Kg |
| --- | --- |
| L-Alanine | 3.5 |
| L-Arginine HCl | 12.1 |
| L-Asparagine | 6.0 |
| L-Aspartic Acid | 3.5 |
| L-Cystine | 3.5 |
| L-Glutamic Acid | 40.0 |
| Glycine | 23.3 |
| L-Histidine HCl-H2O | 4.5 |
| L-Isoleucine | 8.2 |
| L-Leucine | 11.1 |
| L-Lysine HCl | 18.0 |
| **L-Methionine** | 8.2 |
| L-Phenylalanine | 7.5 |
| L-Proline | 3.5 |
| L-Serine | 3.5 |
| L-Threonine | 8.2 |
| L-Tryptophan | 1.8 |
| L-Tyrosine | 5.0 |
| L-Valine | 8.2 |
| Sucrose | 351.68 |
| Corn Starch | 150.0 |
| Maltodextrin | 150.0 |
| Soybean Oil | 80.0 |
| Cellulose (fiber) | 30.0 |
| Mineral Mix, AIN-93M-MX (TD 94049) | 35.0 |
| Calcium Phosphate, monobasic | 8.2 |
| Vitamin Mix, AIN-93-VX (TD 94047) | 13.0 |
| **Choline Bitartrate** | 2.5 |
| TBHQ (antioxidant) | 0.02 |
