# Supplementary material for: Requirement of RIZ1 for Cancer Prevention by Methyl-Balanced Diet
Source: PLoS One. 2008 Oct 13;3(10):e3390. doi: 10.1371/journal.pone.0003390 (PMC2559864; doi:10.1371/journal.pone.0003390)
Supplement: Table S2 — Quantitative RT-PCR and microarray analysis of some histone methyltransferases and related enzymes. Wild type male animals were treated with diet 1 or diet 2 (starting from 3–4 weeks of age) for 1, 2, 4, and 6 months before RNA extraction from livers. Levels of 25 histone methyltransferases and 8 other related enzymes were determined by either quantitative RT-PCR or microarray or both, and the ratio (fold change) Diet 1 versus Diet 2 calculated. Data from quantitative RT-PCR represent means of at least 3 animals per subgroup. *P<0.05 Diet 1 vs. Diet 2 (Student's t-test, 2 tailed). Histone methyltransferases that are not listed here did not show significant expression in the liver as revealed by DNA microarray analysis. Data from microarray analysis represent means of 2 animals per subgroup. DNA microarray analysis revealed downregulation by diet 2 of four small molecule methyltransferases, GNMT, GAMT, NNMT, and TEMT, which were subsequently confirmed by quantitative RT-PCR. (0.08 MB DOC) [file pone.0003390.s002.doc]

| **Diet 1 *vs.* Diet 2** | | | | |
| --- | --- | --- | --- | --- |
| **Months on diet** | **1m** | **2m** | **4m** | **6m** |
| Histone methyltransferases | | | | |
| RIZ1 | 1.1 | 4.2* | 2.3* | 3.1* |
| Prdm3 |  | 1.0 |  |  |
| Prdm9 |  | 1.6 | 1.1 |  |
| Carm1 |  | 1.5 | 0.9 |  |
| Dot1 |  | 1.5 | 1.0 |  |
| Ehmt |  | 1.5 | 1.2 |  |
| Eset |  | 1.4 | 1.1 |  |
| Smyd2 |  | 1.2 | 1.1 |  |
| Suv39H1 |  | 1.4 | 0.9 | 1.1 |
| PR-SET7 |  | 1.1 | 0.8 | 0.7 |
| Prdm1-alpha | 0.4* | 0.2* | 0.2* |  |
| Ezh1 |  | 0.8 | 0.3* |  |
| Ezh2 |  | 0.6 | 0.6 |  |
| Eed (array) |  | 1.0 |  |  |
| Suv4-20H2 (array) |  | 1.3 | 1.0 |  |
| Mll1 (array) |  | 0.8 |  |  |
| Ash1l (array) |  | 1.0 |  |  |
| Smyd1 (array) |  | 0.9 |  |  |
| SET7/9 (array |  | 0.6 |  |  |
| PRDM4 (array |  | 1.1 |  |  |
| G9a (array) |  | 1.0 |  |  |
| G9a |  | 0.9 |  |  |
| Prmt1 (array) |  | 1.0 |  |  |
| Prmt1 |  | 0.9 |  |  |
| Prmt3 (array) |  | 0.7 |  |  |
| Prmt5 (array) |  | 0.7 |  |  |
| Prmt7 (array) |  | 0.9 |  |  |
| Histone demethylases | | | | |
| Jmjd2a |  | 1.1 |  |  |
| Lsd1 |  | 1.3 |  |  |
| DNA methyltransferases | | | | |
| DNMT1 |  | 1.2 |  |  |
| DNMT3A |  | 0.9 |  |  |
| Small molecule methyltransferases | | | | |
| GNMT (array) |  | 3.0 |  |  |
| GNMT | 2.0 | 4.6* |  |  |
| TEMT (array) |  | 3.0 |  |  |
| TEMT | 5.0* | 8.2* |  |  |
| GAMT (array) |  | 1.5 |  |  |
| GAMT |  | 2.3* |  |  |
| NNMT (array) |  | 9.5 |  |  |
| NNMT |  | 4.1* |  |  |
