# Supplementary material for: Requirement of RIZ1 for Cancer Prevention by Methyl-Balanced Diet
Source: PLoS One. 2008 Oct 13;3(10):e3390. doi: 10.1371/journal.pone.0003390 (PMC2559864; doi:10.1371/journal.pone.0003390)
Supplement: Table S5 — Methyltransferases with high Km value for SAM were downregulated by methyl-imbalanced diet. The Km value for SAM and Ki value for SAH are shown for various methyltransferases. The ratio (fold changes) Diet 1 vs. Diet 2 in expression levels of the methyltransferases was calculated from quantitative RT-PCR. Data represent means of at least 3 animals per subgroup at 2 months of diet treatment. *P<0.05 Diet 1 vs. Diet 2 (Student's t-test, 2 tailed). (0.03 MB DOC) [file pone.0003390.s005.doc]

|  | SAM km/SAH ki (μM) | Diet 1 *vs*. Diet 2 |
| --- | --- | --- |
| GNMT | 100/35 | 4.6* |
| GAMT | 49/16 | 2.3* |
| TEMT/INMT | 54/8.6 | 8.2* |
| COMT | 3.1/1.0 | 1.1 |
| TPMT | 3.0/5.8 | 1.0 |
| PCMT | 2.0/0.08 | 1.0 |
| ICMT | 2.1/9.2 | 0.8 |
| DNMT1 | 1.4/1.4 | 1.2 |
| PRMT1 | 8.0/2.3 | 0.9 |
| G9a | 1.8/2.3 | 0.9 |
