# Supplementary figures and images for: Requirement of RIZ1 for Cancer Prevention by Methyl-Balanced Diet
Source: PLoS One. 2008 Oct 13;3(10):e3390. doi: 10.1371/journal.pone.0003390 (PMC2559864; doi:10.1371/journal.pone.0003390)

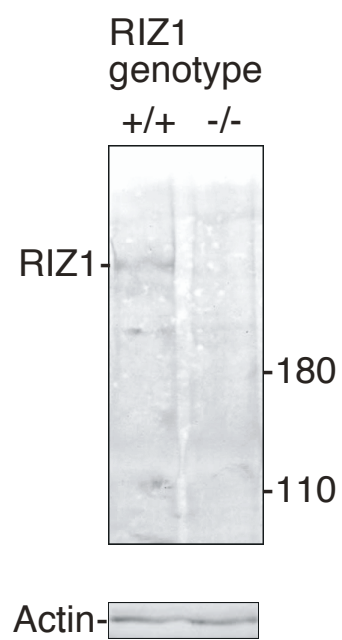

Supplementary Figure 1

Supplement: Figure S1 — Western blot analysis confirming recognition of RIZ1 but not RIZ2 by the Abcam RIZ1-specific antibody ab9710. Total protein extracts of livers from either RIZ1 wild type or knockout animals were resolved by SDS gel followed by western blot using RIZ1 antibody ab9710. Equal loading was confirmed by western blot using beta-actin antibody. (0.07 MB PDF) [file pone.0003390.s006.pdf]

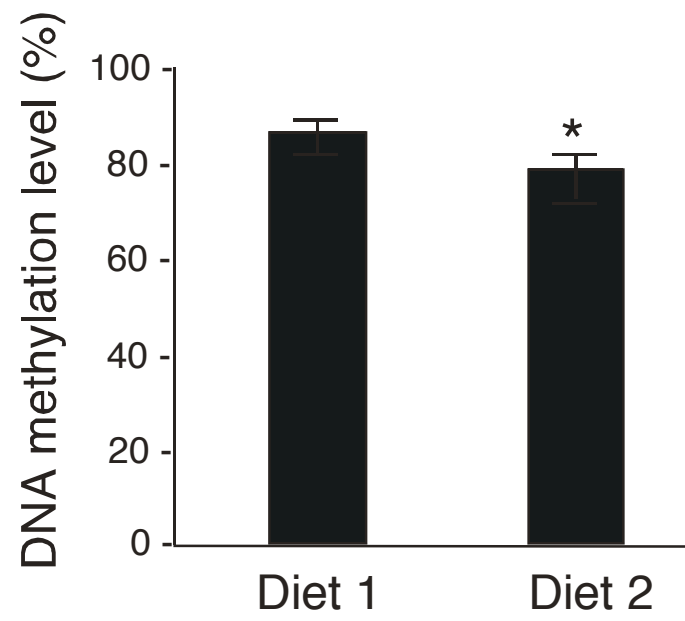

Supplementary Figure 2

Supplement: Figure S2 — DNA hypomethylation in mice fed with diet 2. Total genomic DNAs were isolated from livers of mice on either diet 1 or diet 2 for 15 months. The methylation levels of these DNAs were determined by the Sss1 enzyme assay. Data are the means+SD of 3 animals per subgroup. *P = 0.03 (Student's t-test, 2 tailed). (0.02 MB PDF) [file pone.0003390.s007.pdf]
