# Supplementary material for: Requirement of RIZ1 for Cancer Prevention by Methyl-Balanced Diet
Source: PLoS One. 2008 Oct 13;3(10):e3390. doi: 10.1371/journal.pone.0003390 (PMC2559864; doi:10.1371/journal.pone.0003390)
Supplement: Figure S3 — Analysis of methylation changes in CpG rich regions of the genome by MS-AFLP. A. Genomic DNA isolated from MCF7 cells that were either treated or not treated with the demethylating agent Aza-C were used as a positive control for the MS-AFLP method. B. The genomic DNAs used for analysis were from brain (B) and liver (L) tissues of mice on either diet 1 or diet 2 for 15 months. (1.49 MB PDF) [file pone.0003390.s008.pdf]

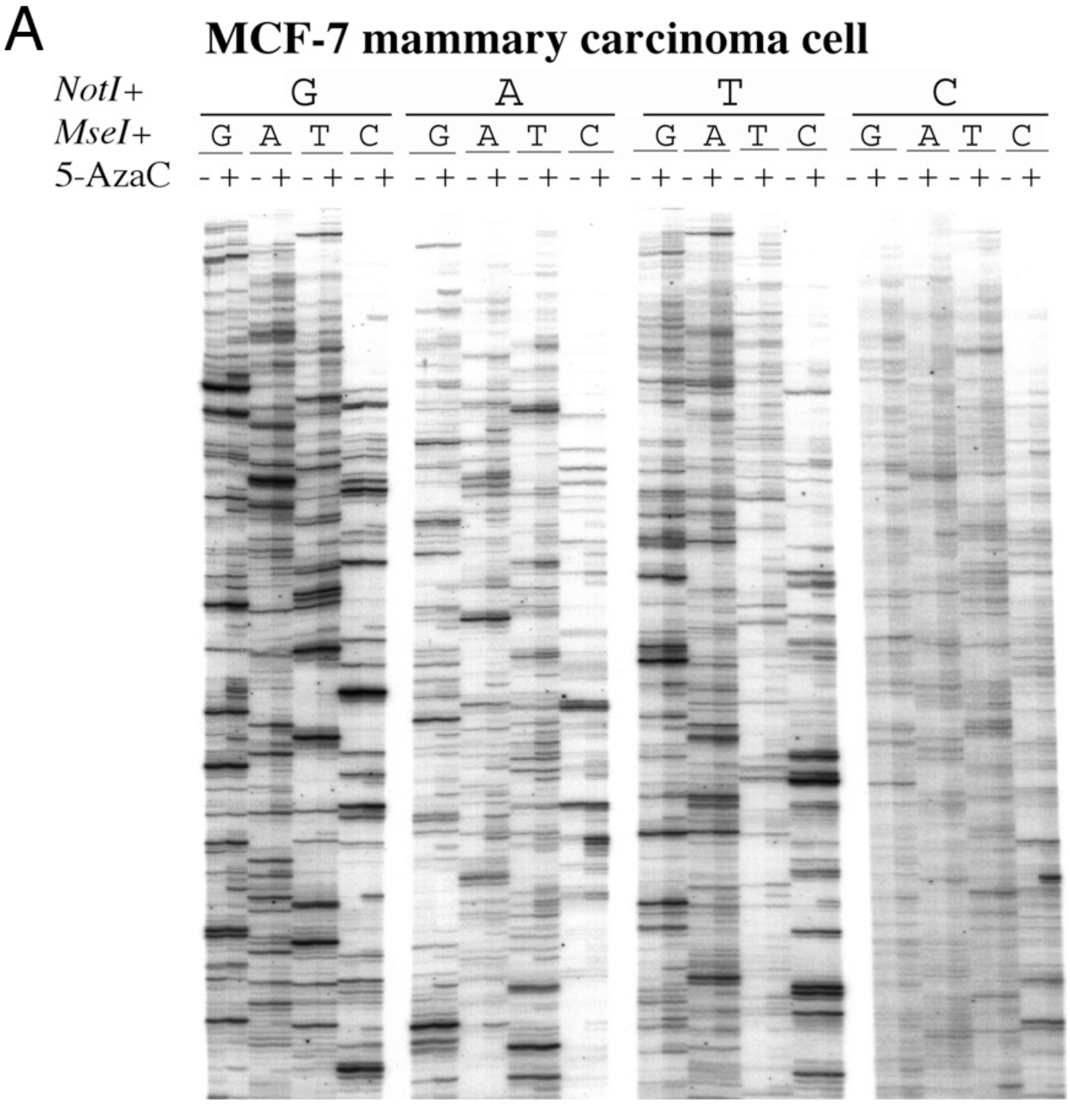
A
MCF-7 mammary carcinoma cell
NotI+
G
A
T
C
MseI+
G A T C
G A T C
G A T C
G A T C
5-AzaC
- + - + - + - +
- + - + - + - +
- + - + - + - +
- + - + - + - +

B

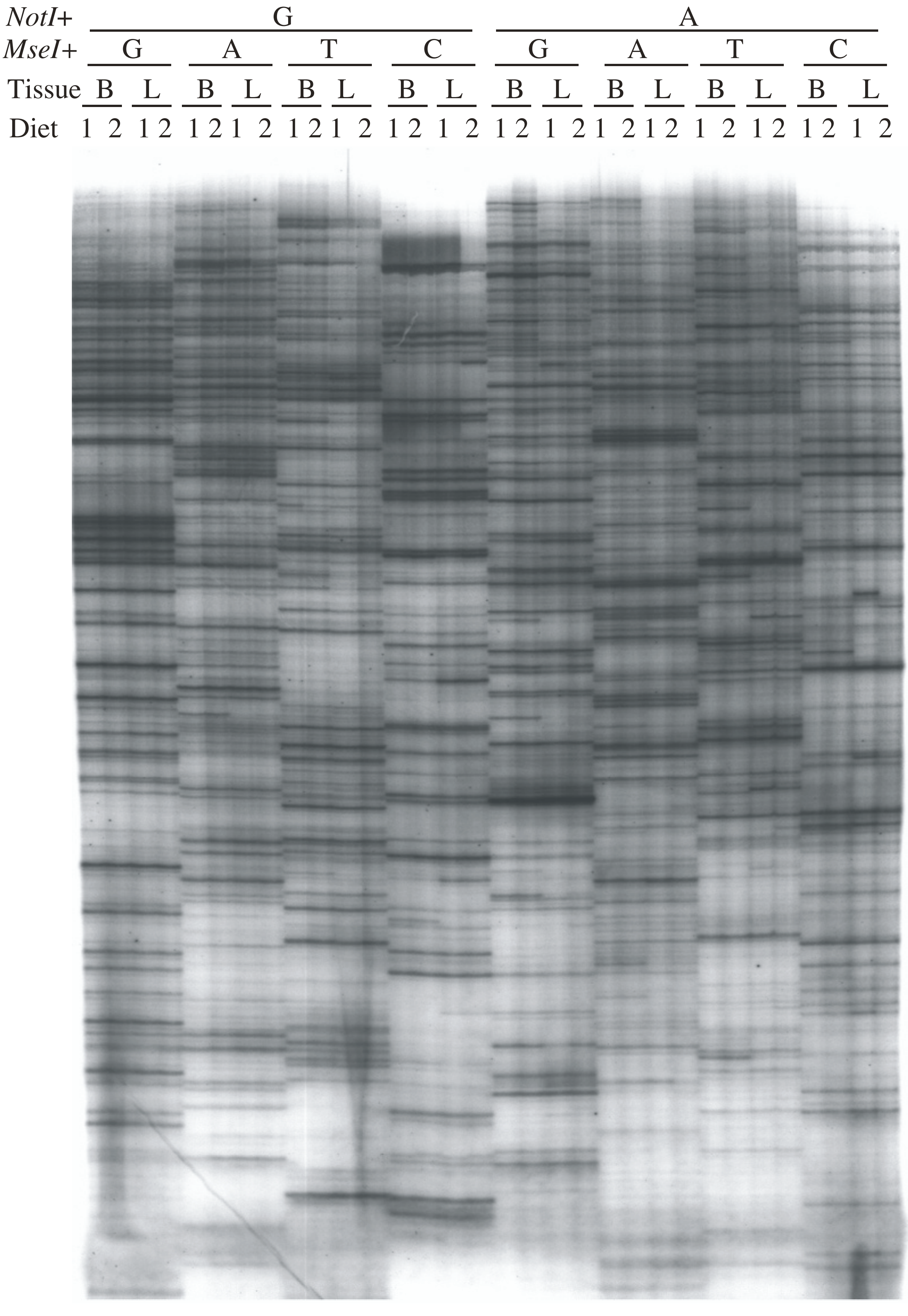
NotI+
G
A
MseI+
G A T C G A T C
Tissue
B L B L B L B L B L B L B L B L
Diet
1 2 1 2 1 2 1 2 1 2 1 2 1 2 1 2 1 2 1 2 1 2 1 2 1 2 1 2 1 2 1 2
